# Effect of Fat Mass Localization on Fat Oxidation During Endurance Exercise in Women

**DOI:** 10.3389/fphys.2020.585137

**Published:** 2020-10-22

**Authors:** Laurie Isacco, Gaël Ennequin, Nathalie Boisseau

**Affiliations:** ^1^EA3920 Prognostic Markers and Regulatory Factors of Cardiovascular Diseases and Exercise Performance Health Innovation Platform, Université Bourgogne Franche-Comté, Besançon, France; ^2^Adaptations Métaboliques à l’Exercice en Conditions Physiologiques et Pathologiques, Centre de Recherche en Nutrition Humaine, Université Clermont Auvergne, Clermont-Ferrand, France

**Keywords:** women, fat mass localization, exercise, lipid utilization, cardio-metabolic risks

## Abstract

Independent of total body fat mass, predominant upper body fat mass distribution is strongly associated with cardio-metabolic comorbidities. However, the mechanisms underlying fat mass localization are not fully understood. Although a large body of evidence indicates sex-specific fat mass distribution, women are still excluded from many physiological studies and their specific features have been investigated only in few studies. Moreover, endurance exercise is an effective strategy for improving fat oxidation, suggesting that regular endurance exercise could contribute to the management of body composition and metabolic health. However, no firm conclusion has been reached on the effect of fat mass localization on fat oxidation during endurance exercise. By analyzing the available literature, this review wants to determine the effect of fat mass localization on fat oxidation rate during endurance exercise in women, and to identify future research directions to advance our knowledge on this topic. Despite a relatively limited level of evidence, the analyzed studies indicate that fat oxidation during endurance exercise is higher in women with lower upper-to-lower-body fat mass ratio than in women with higher upper-to-lower-body fat mass ratio. Interestingly, obesity may blunt the specific effect of upper and lower body fat mass distribution on fat oxidation observed in women with normal weight during endurance exercise. Studying and understanding the physiological responses of women to exercise are essential to develop appropriate physical activity strategies and ultimately to improve the prevention and treatment of cardio-metabolic diseases.

## Introduction

The current global obesity epidemic and related cardio-metabolic comorbidities have resulted in a growing interest in adipose tissue features (Coelho et al., 2013). Besides total body fat mass, fat mass localization is an important and well-established risk factor of metabolic and cardiovascular comorbidities (Fox et al., 2007; Manolopoulos et al., 2010; Smith, 2015). Individuals present different body shapes that are mainly determined by genetic and physiological factors as well as environmental and lifestyle habits (Stefan, 2020). Some people accumulate fat (both subcutaneous and visceral adipose tissue; SAT and VAT, respectively) preferentially in the upper body (android, central, or abdominal distribution), while others predominantly store adipose tissue (SAT) in the lower body (gynoid or peripheral distribution) (Ebbert and Jensen, 2013). These differences provide significant clues to evaluate each individual’s cardiometabolic risk. Jean Vague was the first in 1947 to report a positive association between abdominal obesity and diabetes development (Vague, 1947). Abdominal fat mass, and more specifically VAT, is largely involved in cardio-metabolic disorders, while lower body SAT may have an overall protective effect against morbidity/mortality (Smith, 2015; Stefan, 2020). Currently it is not completely clear why fat mass localizes preferentially in the upper or lower part of the body in some people. However, as select evidence indicates that sex steroid concentrations play a role, it is essential to separately study men and women (Power and Schulkin, 2008; Santosa and Jensen, 2014).

It is recognized that in resting conditions, the upper and lower body fat masses have specific effects on metabolism (e.g., rate of FFA turnover, insulin resistance…). Moreover, the higher lipolysis activity of the upper body fat mass, compared with the lower body fat mass, may differentially influence substrate metabolism (i.e., FFA release and utilization) (Jensen, 1997). While endurance exercise is one of the major factors that influence metabolic adaptations and specifically fat metabolism, firm conclusions on the effects of fat mass localization-induced specific metabolic responses during endurance exercise in women remain to be drawn. Yet, this relationship should be clarified for developing realistic and appropriate training programs to improve women’s health. In this context, this review wanted to determine the effect of fat mass localization on fat oxidation during endurance exercise in this specific population.

## Fat Mass Localization and Fat Oxidation During Endurance Exercise in Women

Regular endurance exercise promotes weight control and is an effective way to counteract cardio-metabolic alterations, particularly by increasing fat oxidation (Melanson et al., 2009a; Thompson et al., 2012; Vissers et al., 2013). Indeed, it is acknowledged that exercise triggers the release of soluble factors (i.e., myokines, hepatokines, osteokines, adipokines) that are involved in the regulation of substrate metabolism and weight control (Gonzalez-Gil and Elizondo-Montemayor, 2020). It has been demonstrated that the nature, amount and intensity of physical activity can influence fat mass localization and/or metabolism through substrate mobilization and oxidation (Mauriège et al., 1997; Maillard et al., 2018). In turn, understanding how fat mass localization may influence fat oxidation during acute and chronic endurance exercise is important for identifying the body shape phenotypes in pre- and post-menopausal women that are resistant to fat utilization and are, therefore, linked to higher risk of obesity and cardio-metabolic complications. This could help to optimize the choice of exercise modalities.

### Premenopausal Women

#### Acute Exercise

This review focused on studies that compared endurance exercise–induced fat oxidation in women with upper or lower body fat depot phenotypes and an actual lack of consensus is observed. Two studies (Isacco et al., 2013, 2014) by the same group reported a decreased ability to oxidize fat during endurance exercise in women with higher upper-to-lower-body fat mass ratio (i.e., the ratio between abdominal fat mass and lower body fat mass). On the other hand, other studies observed only a slight or no difference (Buemann et al., 1994; Swan and Howley, 1994; Kanaley et al., 2001b; van Aggel-Leijssen et al., 2001) (Table 1). This discrepancy could be partly attributed to methodological specificities. First, although all studies included only premenopausal women, the weight status differed (from normal weight to overweight/obesity). Second, the technique used to determine fat mass localization varied among studies (i.e., waist-to-hip ratio, magnetic resonance imaging, dual-energy X-ray absorptiometry). Third, although most studies investigated the impact of fat mass localization on substrate metabolism during acute endurance exercise, other publications focused on exercise training. Fourth, exercise differed in terms of modality, and especially in intensity and duration, which are two major factors influencing substrate oxidation (Romijn et al., 1993; Brooks and Mercier, 1994; Van Loon et al., 2001; Hargreaves and Spriet, 2020). Finally, when specified, the participants’ nutritional status before exercise was not similar among studies (i.e., fasting vs. post-prandial state).

**TABLE 1:** Influence of fat mass localization on fat oxidation during endurance exercise in women.

Authors and publication date	*n*	Age of women (y)	Weight status	Body fat mass localization assessment	Exercise	Substrate metabolism parameters	Results
Isacco et al., 2013	21	22.0 ± 0.6	Women with normal weight (11 women with lower upper-to-lower-body fat mass ratio and 10 women with higher upper-to-lower-body fat mass ratio)	DXA	Acute; cycling at 65% VO_2__max_ for 45 min (3 h after a standardized meal)	RER, fat and CHO oxidation rates, plasma glycerol, FFA, glucose, insulin concentrations	Women with lower upper-to-lower-body fat mass ratio:↑ fat mobilization and oxidation compared with women with higher ratio
Isacco et al., 2014	21	22.0 ± 0.6	Women with normal weight (11 women with lower upper-to-lower-body fat mass ratio and 10 women with higher upper-to-lower-body fat mass ratio)	DXA	Acute; submaximal incremental exercise to determine Fat_max_ and maximal lipid oxidation rates (fast)	RER, fat and CHO oxidation rates, plasma glycerol, FFA, glucose, insulin concentrations	Women with lower upper-to-lower-body fat mass: ↑ maximal fat oxidation rates compared with women with higher ratio
Swan and Howley, 1994	20	? Premenopausal	10 women with lower body obesity and 10 women with upper body obesity	WHR	Acute; walk bouts at 55–60% VO_2__max_ for 60 min	RER, fat oxidation rates	→ Fat oxidation during exercise between women with upper and lower body obesity
van Aggel-Leijssen et al., 2001	21	32.8 ± 9.6	8 women with lower body obesity and 13 women with upper body obesity	WHR	Training; 12 weeks of cycling at ∼40% VO_2__max_ for ∼ 60 min, 3 times per week Fat oxidation rate measurement: cycling at 50% VO_2__max_ for 1 h	RER, fat oxidation rates, plasma glycerol, FFA, glucose, insulin concentrations	Before training: → After training: RER ↓and fat oxidation rates ↑ in women with upper body obesity compared with women with lower body obesity; → fat metabolism in women with lower body obesity
Kanaley et al., 2001b	31	35.0 ± 7.0 (women with normal weight) and 32.2 ± 1.7 (women with obesity)	8 women with normal weight and 23 women with obesity (11 women with lower body obesity and 12 women with upper body obesity)	WC	Acute; treadmill at 70% VO_2__max_ for 30 min (fast) Training; 16 weeks of aerobic training at ∼70% VO_2__max_ for 40 min, 3 times per week	RER, fat and CHO oxidation rates	Before training: →fat oxidation between women with upper and lower body obesity After training: ↑ CHO oxidation and →fat oxidation compared with pre-training Data on women with upper and lower obesity were pooled
Buemann et al., 1994	34	32.5 ± 2.5 (women with upper body obesity) and 36.1 ± 2.8 (women with lower body obesity)	19 women with upper body obesity and 15 women with lower body obesity	WHR	24 h in metabolic chamber with a predetermined physical activity program (3*10 min of cycling at 75 watts and 25*3.5 m of walking in one time)	RER; no specific analysis for physical activity	→ Substrate oxidation rates during 24 h

*CHO, carbohydrates; DXA, dual-energy X-ray absorptiometry; Fat_*max*_, maximal fat oxidation rates; FFA, free fatty acids; MRI, magnetic resonance imaging; RER, respiratory exchange ratio; VO_2__*max*_, maximal oxygen consumption; WC, waist circumference; WHR, waist-to-hip ratio. ↑, increase; ↓, decrease; →, similar; ?, not known.*

**FIGURE 1:**
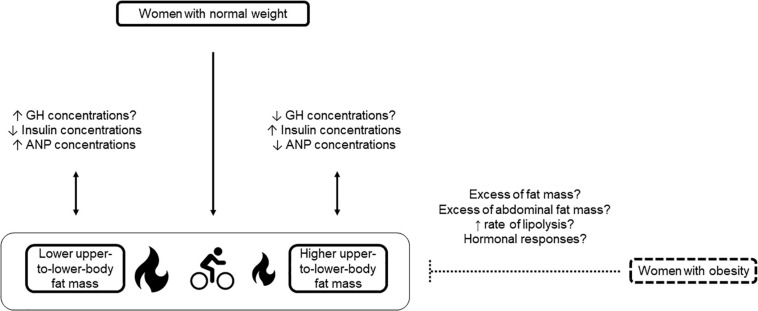
Influence of fat mass localization on fat oxidation during acute endurance exercise in premenopausal women with normal weight and obesity. At the same relative endurance exercise intensity, women with normal weight and lower upper-to-lower-body fat mass oxidize more fat than women with normal weight and higher upper-to-lower-body fat mass. Decreased insulin concentrations and increased ANP concentrations in women with lower upper-to-lower-body fat mass compared with women with higher upper-to-lower-body fat mass may partly explain this result. Data on GH need to be confirmed. In women with obesity, no difference is observed in fat oxidation during acute endurance exercise in function of the body shape. In women with obesity, excess fat mass and/or abdominal fat mass and high rate of lipolysis may blunt the impact of fat mass localization on fat oxidation during acute endurance exercise. Future studies should investigate the specific hormonal responses in women with upper and lower obesity. ANP, atrial natriuretic peptide; GH, growth hormone; ↑, increased; ↓, decreased; ?, to be confirmed. 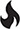 Lower fat oxidation;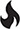 Higher fat oxidation; 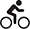 Endurance exercise.

Yet, some trends can be detected. In women with obesity, the effect of excess total body fat mass may predominate over the effect of its localization (Figure 1). Indeed, like in men (Numao et al., 2006), no difference was observed in fat oxidation during exercise between women with upper (i.e., fat mass deposition predominantly in the abdominal region) and lower body obesity (i.e., fat mass deposition predominantly in the lower body) (Swan and Howley, 1994; Kanaley et al., 2001b; van Aggel-Leijssen et al., 2001). Interestingly, results were similar in these studies, despite disparities in terms of exercise intensity and duration (i.e., from 40 to 70% of VO_2__max_ and for 40 to 60 min). Haufe and collaborators found that exercise-induced fat oxidation is not associated with fat mass localization (Haufe et al., 2010). However, this large study enrolled men and women without any sex-specific analysis. Horowitz and collaborators observed that women with upper body obesity use more fat as fuel than women with normal weight during endurance exercise, likely due to an increase in intramyocellular triglyceride utilization (Horowitz and Klein, 2000). Finally, despite greater FFA turnover in women with upper than with lower body obesity, the respiratory quotient and thus substrate oxidation were comparable in these two groups during 24 h (Buemann et al., 1994). Although these results are not specific to exercise, it is worth noting that the 24-h indirect whole-body calorimetry investigation integrated a predetermined physical activity program. Overall, it may be hypothesized that individuals with obesity are prone to increased abdominal adipose tissue, regardless of their body shape, and that the transient rise in lipolytic activity induced by exercise cannot override and/or modify the chronically high lipolysis rate observed in obesity (Figure 1).

Results in women with normal weight are different. We reported that fat mass localization influences fat oxidation during cycling (65% of VO_2__max_ for 45 min) (Isacco et al., 2013). Women with lower upper-to-lower-body fat mass ratio showed greater fat mobilization and oxidation during exercise compared with women with higher upper-to-lower-body fat mass ratio (Figure 1). We suggested that the higher plasma levels of growth hormone and ANP and the reduced insulin concentration in women with lower upper-to-lower-body fat mass ratio could explain these between-group differences. Indeed, catecholamines, ANP, growth hormone and insulin are important regulators of lipid mobilization and also of fat utilization, due to the association between plasma FFA concentration and oxidation level (Buemann et al., 1994; Jocken and Blaak, 2008). Catecholamines and ANP (and growth hormone to a lesser extent) act as lipolytic hormones, while insulin is the main anti-lipolytic hormone. Insulin favors fat storage in adipose tissue by enhancing glucose uptake and lipogenesis, and by inhibiting lipolysis. The lipolytic effect of catecholamines is determined by the ratio between lipolytic (β-adrenoreceptors) and anti-lipolytic (α-adrenoreceptors) receptors. Interestingly, ANP exercises a lipolytic action through an independent pathway (cyclic guanosine monophosphate and protein kinase G) from the signaling cascade regulated by catecholamines and insulin (cyclic adenosine monophosphate and protein kinase A) (Sengenes et al., 2000; Jocken and Blaak, 2008; Stinkens et al., 2015).

In resting condition, growth hormone concentrations were not different between groups and the minimal growth hormone-induced lipid mobilization during exercise suggested a negligible effect. Interestingly, while glucose concentrations were not different between groups, women with higher upper-to-lower-body fat mass ratio exhibited higher post-prandial insulin levels, indicating an insulin resistance risk. The significant difference in ANP concentrations at rest and during exercise suggests a specific regulation of ANP in function of body shape (Isacco et al., 2013; Isacco and Boisseau, 2016) (Figure 1). It appears relevant to carry out additional clinical and cellular studies on this issue to facilitate phenotyping and cardio-metabolic risk management in women with normal weight. These results were obtained using exercise modalities with specific duration and intensity that are two major factors influencing substrate oxidation (Romijn et al., 1993; Brooks and Mercier, 1994; Van Loon et al., 2001; Hargreaves and Spriet, 2020).

In addition, the lower metabolic flexibility in women with higher upper-to-lower-body fat mass ratio increases their risk of cardio-metabolic alterations, particularly insulin resistance (Rynders et al., 2018). Similarly, analysis of the maximal fat oxidation rates during a specific exercise protocol showed that the maximal fat oxidation rates elicited at higher exercise intensity are higher in women with lower upper body fat mass than in women with higher upper body fat mass (Isacco et al., 2014). Altogether, these findings indicate that in women with normal weight, fat mass localization should be taken into account to identify women at higher risk of cardio-metabolic diseases and to recommend adapted exercise protocols (Isacco and Miles-Chan, 2018).

#### Exercise Training

Endurance exercise has many health benefits, including on body weight and composition (Donnelly et al., 2009). Endurance training, associated with a balance diet, promotes a shift in fat oxidation during exercise by increasing mitochondrial density and respiratory function, by reducing muscle glycogen utilization, and by decreasing catecholamine and lactate levels during steady state exercise. Moreover, endurance training decreases the activity of α-adrenergic receptor, and increases the activity of β-adrenergic receptor, the number of FFA transporters, the content of fatty acid transport protein, the enzymatic activity of the Krebs cycle, the β-oxidation pathway and the components of the electron transport chain to oxidize FFA (Brooks and Mercier, 1994; Holloszy and Kohrt, 1996; Talanian et al., 2010; Yoshida et al., 2013).

Interestingly, while no difference was observed between women with upper and lower body obesity at baseline, low-intensity exercise training (cycling at ∼40% VO_2__max_ for about 60 min, 3 times per week for 12 weeks) increased the relative fat oxidation rates during exercise only in women with upper body obesity (van Aggel-Leijssen et al., 2001). It is difficult to explain this finding and the authors emphasized that the greater ability to oxidize fat following exercise training in women with upper body obesity was likely due to an increase in intramyocellular triglycerides and very low-density lipoprotein triglycerides rather than in FFA oxidation (adipose tissue lipolysis). Moreover, they suggested that after exercise training, fat may be more readily mobilized from the upper than the lower body fat mass depot in women with obesity (van Aggel-Leijssen et al., 2001). On the other hand, exercise training at higher intensity, but still in the light- to-moderate-intensity range that enables maximal lipid oxidation rates, could favor fat utilization in women with lower body obesity. Indeed, it was previously observed that the lipolysis rate at rest is increased in women with upper body obesity compared with those with lower body obesity (Jensen et al., 1989; Martin and Jensen, 1991). Therefore, it could be hypothesized that the exercise intensity threshold to promote lipolysis and fat oxidation is different for women with upper and lower body obesity. However, after 16 weeks of endurance training (70% of VO_2__max_ for 30 min, 3 times per week), no difference in fat oxidation rates during exercise was observed compared with pre-training in women with obesity (Kanaley et al., 2001b). It is worth noting that in this study, the data of women with upper and lower body obesity (*n* = 5/each) were pooled, and this did not allow investigating the impact of fat mass localization on exercise training-induced fat oxidation. In addition, training intensity (70% of VO_2__max_) might have been too high for assessing optimal fat oxidation. To our knowledge, no information is available on the impact of exercise training on substrate oxidation in relation with fat mass localization in women with normal weight.

Interestingly, Van Aggel-Leijssen and colleagues found that the relative fat oxidation during exercise increased only in women with upper obesity, and they did not observe any change in body weight and composition in both groups (women with upper and lower obesity) after the 12 weeks of endurance training. These results question the influence on body composition of the increased fat oxidation in response to endurance training in this population. Indeed, endurance exercise increases the capacity to use fat at rest and during exercise, suggesting an effect on body weight and fat mass loss via greater fat oxidation (Jeukendrup, 2002). However, higher fat oxidation during exercise and changes in body composition in response to exercise training are not necessarily associated. Indeed, due to the effect of carbohydrate ingestion on fat metabolism, the pre-exercise nutritional status (fasting vs. post-prandial) and eating habits (quality and quantity) must be considered when studying body weight and fat mass loss (Melanson et al., 2009b). In addition, the magnitude of fat oxidation during exercise may not be sufficient to induce fat mass loss. The potential compensation in fat oxidation during the subsequent hours/days (e.g., post-exercise period, meals, sleep) should also be taken into account.

Nevertheless, even if increased fat oxidation may not be associated with a decrease in fat mass in response to endurance training, the exercise-mediated improvement in fat oxidation is important not only for body composition and weight management, but also for cardio-metabolic health. Indeed, the capacity to oxidize fat during exercise is inversely related to cardio-metabolic comorbidities (e.g., insulin resistance) and to the progression of metabolic diseases (Brooks et al., 2004; Kelley, 2005). Therefore, it is essential to promote additional studies on this topic considering both components of fat balance.

### Post-menopausal Women

It is recognized that aging is associated with increased fat mass accumulation and menopause leads to a shift toward upper body fat mass deposition. However, and surprisingly, little is known about the effect of fat mass localization on substrate oxidation during endurance exercise in post-menopausal women. Some studies investigated the influence of menopause and the related body composition modifications on substrate metabolism at rest and during exercise (Lovejoy et al., 2008; Abildgaard et al., 2013), but none considered fat oxidation during exercise in relation with the specific post-menopausal body shape.

It has been reported that in women with normal weight, whole-body lipolysis is not affected by menopause in post-absorptive and also in hyperinsulinemic conditions (Toth et al., 2002). Lipolysis is higher in abdominal than in peripheral adipocytes in post-menopausal women with upper and also lower body obesity (Nicklas et al., 1996). In addition, in post-menopausal women with obesity, higher VAT is associated with increased fat oxidation, independent of total body fat mass (Nicklas et al., 1995). According to these results, obesity may override the effect of body shape on lipolysis, while fat oxidation depends on fat mass localization. It is worth noting that many studies that investigated the effect of menopause on lipid metabolism were performed in women with obesity, mainly due to its increased prevalence within this population. It would be relevant to know whether results are similar in women with normal weight and whether the obesity history (onset before vs. after menopause) leads to distinct lipid metabolism responses.

As weight gain in menopause increases the risk of obesity and cardio-metabolic disorders, many women may want to lose weight. Hypocaloric diets induce fat mass loss in the short term, but the rate of weight loss progressively decreases over time. The metabolic adaptations occurring during prolonged diet restriction (i.e., decline in resting metabolic rate, lipolytic activity, fat oxidation) might explain the reduced weight loss rate and the predisposition to weight regain when individuals return to a normocaloric diet (Schutz et al., 1992; Ravussin and Swinburn, 1993; Reynisdottir et al., 1995; Nicklas et al., 1997). In post-menopausal women with obesity, endurance training counteracts this decline in weight loss (Nicklas et al., 1997). These results suggest the importance of regular exercise to minimize the potential adverse effects of menopause associated with obesity on lipid metabolism. Indeed, physical activity is a key component of menopause management (Kanaley et al., 2001a). Lange and colleagues reported that lipolysis in subcutaneous abdominal adipose tissue during endurance exercise is not altered in post-menopausal women (Lange et al., 2002). Interestingly, it has been observed that fat oxidation during exercise is reduced in post-menopausal compared with premenopausal women. This difference was mainly explained by the reduced lean body mass, suggesting, once more, the importance of regular exercise to manage body composition (Abildgaard et al., 2013). In addition, the lower ability to oxidize fat was related to the worsened metabolic profile and increased VAT, thus highlighting the influence of fat mass localization on substrate oxidation during exercise in post-menopausal women. Comparison of the substrate oxidation rates during cycling (50% VO_2__max_ for 40 min) in men with obesity and post-menopausal women with obesity showed that while upper body fat mass was not significantly different between men and women, VAT was lower and abdominal SAT and fat oxidation rates were higher in women than in men. This suggests the importance of upper adipose tissue quality for substrate metabolism. The authors noted that sexual dimorphism in substrate oxidation during exercise was unlikely to be explained by plasma estrogen concentrations, which were comparable between groups. They hypothesized that intramyocellular triglyceride content and muscle morphology may play a role (Numao et al., 2009). Interestingly, substrate oxidation during endurance exercise (80% VO_2__max_ for 30 min) is comparable in post-menopausal women undergoing or not hormone replacement therapy; however, hormone replacement therapy is associated with greater reliance on fat proportional with exercise duration (Johnson et al., 2002). Prolonged exercise and/or lower exercise intensity might favor fat oxidation in post-menopausal women on hormone replacement therapy, mainly due to the direct and indirect effects of synthetic estrogens on lipid metabolism.

Overall, more randomized clinical trials are needed to investigate the effect of fat mass localization on fat oxidation during acute and chronic endurance exercise in women with normal weight and obesity, before and after menopause. In addition, due to the lack of experimental studies, the present review only focused on fat oxidation during endurance exercise and it is not known whether these findings might also apply to other exercise modalities (e.g., arm cycling, intermittent exercise, resistance exercise), also relevant for the prevention and treatment of cardio-metabolic diseases (Ormsbee et al., 2009; Davitt et al., 2013; Petridou et al., 2019).

## Conclusion

Over the years, many studies have described, mainly at rest, the deleterious effects of upper body adiposity and its association with the risk of cardio-metabolic alterations. Although it is acknowledged that regular physical activity plays a pivotal role in fat metabolism regulation, and thus in body composition management and cardio-metabolic health, data on the impact of fat mass localization on substrate utilization during exercise in women are scarce.

Interestingly, although few studies are available on this topic, the weight status appears to be a confounder in the relationship between fat mass localization and fat oxidation during acute endurance exercise in premenopausal women (Figure 1). Higher abdominal fat depot is associated with impaired submaximal and maximal fat oxidation and metabolic inflexibility during exercise in women with normal weight. Conversely, no difference is observed in women with upper and lower body obesity. Understanding these disparities is essential to provide optimized prevention and treatment strategies for cardio-metabolic comorbidities. Also, researchers and practitioners should pay more attention to the women’s characteristics to avoid misconceptions and to elaborate adapted intervention strategies.

## Author Contributions

LI and NB had the idea for the review article. LI and GE performed the literature search and data analysis and drafted the review article. NB critically revised it. All authors contributed to the article and approved the submitted version.

## Conflict of Interest

The authors declare that the research was conducted in the absence of any commercial or financial relationships that could be construed as a potential conflict of interest.

## Abbreviations

ANP: atrial natriuretic peptide
FFAs: free fatty acids
SAT: subcutaneous adipose tissue
VAT: visceral adipose tissue.
